# Systemic inflammatory response syndrome in patients with severe fever with thrombocytopenia syndrome: prevalence, characteristics, and impact on prognosis

**DOI:** 10.1186/s12879-024-09026-4

**Published:** 2024-01-30

**Authors:** Zhongwei Zhang, Xue Hu, Qunqun Jiang, Fangzhou Jiao, Qian Du, Jie Liu, Mingqi Luo, Anling Li, Liping Deng, Yong Xiong

**Affiliations:** 1 Department of Infectious Disease, Zhongnan Hospital of Wuhan University, Wuhan, China; 2 Department of Infectious Diseases, Tongji Hospital, Tongji Medical College and State Key Laboratory for Diagnosis and Treatment of Severe Zoonotic Infectious Disease, Huazhong University of Science and Technology, Wuhan, China; 3 Department of Clinical Laboratory, Center for Gene Diagnosis, and Program of Clinical Laboratory Medicine, Zhongnan Hospital of Wuhan University, Wuhan, China

**Keywords:** Severe fever with thrombocytopenia syndrome, Systemic inflammatory response syndrome, Prevalence, Clinical characteristics, Prognosis

## Abstract

**Background:**

Severe fever with thrombocytopenia syndrome (SFTS) is an emerging zoonosis with a high fatality rate in China. Previous studies have reported that dysregulated inflammatory response is associated with disease pathogenesis and mortality in patients with SFTS. This investigation aimed to evaluate the prevalence and characteristics of systemic inflammatory response syndrome (SIRS), and its impact on prognosis.

**Methods:**

Data on demographic characteristics, comorbid conditions, clinical manifestations, laboratory parameters, and survival time of patients with SFTS were collected. Patients were divided into the non-SIRS and SIRS groups according to the presence of SIRS, then their clinical data were compared.

**Results:**

A total of 290 patients diagnosed with SFTS were retrospectively enrolled, including 126(43.4%) patients with SIRS. Patients in the non-survivor group had more prevalence of SIRS than patients in the survivor group (*P* < 0.001), and SIRS (adjusted OR 2.885, 95% CI 1.226–6.786; *P* = 0.005) was shown as an independent risk factor for prognosis of patients with SFTS. Compared with patients without SIRS, patients with SIRS had lower WBC and neutrophils counts, and fibrinogen levels, but higher AST, LDH, amylase, lipase, CK, CK-MB, troponin I, APTT, thrombin time, D-dimer, CRP, IL-6, SAA levels, and viral load. The cumulative survival rate of patients with SIRS was significantly lower than that of patients without SIRS. Patients with SIRS also showed a higher incidence of bacterial or fungal infections than patients without SIRS.

**Conclusions:**

SIRS is highly frequent in patients with SFTS, and it is associated with high mortality.

**Supplementary Information:**

The online version contains supplementary material available at 10.1186/s12879-024-09026-4.

## Introduction

Severe fever with thrombocytopenia syndrome (SFTS) is an emerging tick-borne zoonosis caused by SFTS virus (SFTSV), which was initially reported in rural areas of China in 2009 [1–3]. SFTSV is mainly transmitted by tick bites and can be transmitted among humans. Additionally, direct contact with the body fluids of infected dogs and cats can also lead to SFTSV infection in humans, even aerosol-containing SFTSV is a potential transmission route, which is recognized

as a critical public health problem [4–6].

Patients with SFTS have diverse clinical manifestations, it usually presents with abrupt onset of fever and thrombocytopenia, accompanied by some nonspecific symptoms such as anorexia, dizziness, headache, nausea, vomiting, and diarrhea, while patients with serious SFTS can develop multiple organ dysfunction syndrome, with a reported mortality rate ranging from 6.3% to 30% in various epidemic areas [7–10]. The extensive distribution, high morbidity and mortality of SFTS make it a significant newly emerging infectious disease in China. Therefore, it is urgent to identify the population with poor prognosis and provide intensive treatment as soon as possible to decrease the mortality of SFTS patients.

Systemic inflammatory response syndrome (SIRS) is a complex pathophysiologic response to a strike such as infection, trauma, burns, pancreatitis, or a variety of other damages [11]. Previous studies have demonstrated that multiple cytokines are produced during the acute phase of SFTSV infection, leading to a dysregulated inflammatory response, which is associated with disease severity and mortality in SFTS patients [12, 13]. A retrospective study has revealed that SIRS score can be used to establish risk models for mortality of SFTS patients [14]. Thus, a deep comprehension of the clinical characteristics of SIRS and its associated outcomes is important for providing intensive personalized medical interventions to improve the prognosis of patients with SFTS. However, until recently, the data for SIRS in SFTS were limited.

The primary objective of our study was to explore the prevalence and clinical features of SIRS and to study its impact on outcomes in patients with SFTS. We also investigate the relationship between SIRS and bacterial or fungal infections.

## Patients and methods

### Patients

A total of 290 consecutive patients diagnosed with SFTS admitted to the Department of Infectious Disease, Zhongnan Hospital of Wuhan University between August 2016 and July 2023 were enrolled into a retrospective cohort. The patients were then divided into the non-SIRS (*n* = 164) and SIRS groups (*n* = 126) according to the presence of SIRS. Among these patients, 94 patients were diagnosed with SIRS at admission, and 32 patients were diagnosed with SIRS during hospitalization.

### Diagnostic criteria

The criteria for diagnosing SFTS were as follows [7]: febrile patients (temperatures of 37.3 ˚C or higher for over 24 h) and decreased platelet count; laboratory-confirmed SFTSV infection by detection of viral RNA in serum via reverse transcriptase polymerase chain reaction.

The diagnosis of SIRS was made according to the recommendations of the American College of Chest Physicians/Society of Critical Care Medicine Consensus Conference [15]. Diagnosis of SIRS met at least two of the following criteria: (1) temperature of > 38 ˚C or < 36 ˚C; (2) heart rate of > 90 beats/minute; (3) respiratory rate of > 20 breaths/minute; (4) white blood cell (WBC) count > 12,000/mm^3^ or < 4000/mm^3^, or differential count > 10% immature polymorphonuclear neutrophil cells.

Bacterial infections were diagnosed through a combination of clinical features, laboratory tests, and imaging findings. Bloodstream infections and urinary tract infections were diagnosed by the growth of bacteria or fungi in cultured blood or urine samples. Pulmonary infections were diagnosed by the presence of respiratory symptoms and pulmonary infections evidenced by chest X-ray or computed tomography, accompanied by positive sputum culture. Fungal infections were defined as follows. Invasive candidiasis: isolation of *Candida spp* in one or more blood cultures or from other sterile body fluids. Invasive aspergillosis: detection of *Aspergillus* by direct examination and/or culture of respiratory samples in the presence of radiological imaging compatible with pulmonary infections [16]. The timing of diagnosing bacterial or fungal infections was the time point at which SIRS occurred.

Patients were excluded if they fulfilled one or more of the following reasons: (1) age ≤ 18 years or ≥ 80 years, (2) the presence of preterminal comorbidities (heart disease New York Heart Association III–IV, severe chronic obstructive pulmonary disease, chronic renal failure), (3) any other types of immunodeficiency, (4) history of malignant tumor, (5) incomplete clinical data or missed follow-up.

### Data collection

The medical data of patients were collected, demographic characteristics, comorbid conditions, clinical manifestations, radiological findings and laboratory test results including white blood cell (WBC) count and percentage, neutrophils count and percentage, lymphocyte count and percentage, platelet count, hemoglobin, alanine aminotransferase (ALT), aspartate aminotransferase (AST), total bilirubin (TBIL), albumin, globulin, alkaline phosphatase (ALP), gamma glutamyl transpeptidase (GGT), lactate dehydrogenase (LDH), amylase, lipase, blood urea nitrogen (BUN), creatinine, cystatin-C, sodium, potassium, creatinine kinase (CK), creatinine kinase myocardial b fraction (CK-MB), troponin I, brain natriuretic peptide (BNP), prothrombin time (PT), international normalized ratio (INR), prothrombin activity (PTA), activated partial thromboplastin time (APTT), thrombin time (TT), fibrinogen, D-dimer, C-reactive protein (CRP), procalcitonin, interleukin-6 (IL-6), serum amyloid A (SAA), erythrocyte sedimentation rate (ESR), SFTSV viral load and survival time were collected. The presence of bacterial or fungal infections, acute kidney injury (AKI), acute respiratory distress syndrome (ARDS), and shock on admission and during hospitalization was also recorded.

### Ethics, consent and permissions

The study was conducted according to the principles expressed in the Declaration of Helsinki and approved by the Ethics Committee of Zhongnan Hospital of Wuhan University (no. 2023117 K).

### Statistical analysis

Categorical variables were expressed as numbers (percentages) and were compared by the Chi-square test or Fisher’s exact test. Continuous variables were expressed as the means ± standard deviations for data with a normal distribution or medians with interquartile ranges (P25-P75) for data with a non-normal distribution, which were compared by the Student’s t-test or Mann–Whitney U test, respectively. The cumulative survival rates of patients were evaluated using the Kaplan-Meier method and were compared by the Log-rank test. Univariate and multivariate logistic regression analyses were performed to investigate risk factors for in-hospital mortality of SFTS patients. The collinearity analysis was used to identify the overlapping risk factors for in-hospital mortality, the variance inflation factor (VIF) > 10 or tolerance < 0.1 was a criteria of severe collinearity. All data were analyzed with IBM Corp. (2019) IBM SPSS Statistics for Windows, Version 26.0. IBM Corp., Armonk, and *P* < 0.05 (two-sided) was considered statistically significant.

## Results

### Demographic characteristics, comorbid conditions, clinical manifestations, laboratory test results of SFTS patients in the survivor and non-survivor groups

A total of 290 consecutive patients diagnosed with SFTS were enrolled, including 240 patients in the survivor group and 50 patients in the non-survivor group. Compared with patients in the survivor group, patients in the non-survivor group were older, had lower frequency of male, more presence of sputum, abdominal pain, encephalopathy, SIRS, bacterial or fungal infections, AKI, ARDS, and shock. However, no significant differences were observed in the frequency of diabetes and hypertension, days from onset to admission, as well as the presence of headache, dizziness, cough, chest distress, anorexia, nausea, vomiting, and diarrhea.

Among these laboratory parameters, platelet count, albumin and fibrinogen levels, PTA of patients in the non-survivor group were significantly lower than those of patients in the survivor group. However, patients in the non-survivor group had higher ALT, AST, ALP, GGT, LDH, amylase, lipase, BUN, creatinine, cystatin-C, potassium, CK, CK-MB, troponin I, BNP, PT, INR, APTT, TT, D-dimer, CRP, procalcitonin, IL-6, SAA, ESR, ferritin levels, and viral load than patients in the survivor group. No significant differences were found between the two groups for the remaining variables (Table 1).

**Table 1:** Demographic characteristics, comorbid conditions, clinical representations, laboratory parameters of patients with SFTS between the survivor and non-survivor groups

	Survivor (*n* = 240)	Non-survivor (*n* = 50)	*P* value
Male, n (%)	128(53.3)	19(38.0)	0.049
Age (years)	64 ± 7	66 ± 8	0.048
Diabetes, n (%)	16(6.7)	7(14.0)	0.081
Hypertension, n (%)	56(23.3)	15(30.0)	0.319
Bacterial or fungal infections, n (%)	126(52.5)	49(98.0)	< 0.001
Days from onset to admission	6(5–8)	7(5–9)	0.548
Clinical manifestations, n (%)			
Neurological			
Headache	43(17.9)	12(24.0)	0.318
Dizziness	76(31.7)	19(38.0)	0.385
Encephalopathy	21(8.8)	24(48.0)	< 0.001
Respiratory			
Cough	54(22.5)	13(26.0)	0.495
Sputum	41(17.1)	18(36.0)	0.003
Chest distress	46(19.2)	13(26.0)	0.275
Gastrointestinal			
Anorexia	174(72.5)	38(76.0)	0.612
Nausea	158(65.8)	34(68.0)	0.768
Vomiting	77(32.1)	14(28.0)	0.571
Abdominal pain	25(10.4)	23(46.0)	< 0.001
Diarrhea	56(23.3)	14(28.0)	0.483
SIRS	91(37.9)	35(70.0)	< 0.001
ARDS	6(2.5)	14(28.0)	< 0.001
AKI	35(14.6)	32(64.0)	< 0.001
Shock	8(3.3)	17(34.0)	< 0.001
Laboratory parameters			
WBC (10^9^/L)	3.6(2.0-6.6)	3.7(2.7–6.4)	0.671
Neutrophils (10^9^ /L)	2.1(1.1–4.8)	2.9(1.3–4.9)	0.413
Neutrophils (%)	68.6(52.1–82.0)	71.0(59.0-82.8)	0.492
Lymphocyte (10^9^/L)	0.7(0.5–1.1)	0.6(0.4–1.4)	0.689
Lymphocyte (%)	22.0(11.7–33.7)	21.8(13.5–28.3)	0.484
Platelet (10^9^ /L)	43(31–63)	34(21–56)	0.002
Hemoglobin (g/L)	125 ± 20	119 ± 21	0.211
ALT (U/L)	63(44–108)	138(74–271)	< 0.001
AST (U/L)	143(69–292)	596(282–1151)	< 0.001
TBIL(μmol/L)	11.7(8.7–17.2)	13.8(9.6–22.1)	0.089
Albumin (g/L)	30.3 ± 5.8	27.7 ± 4.2	< 0.001
Globulin (g/L)	25.9(23.4–29.4)	25.8(22.3–30.4)	0.881
ALP (U/L)	71(55–93)	109(73–194)	< 0.001
GGT (U/L)	35(21–82)	90(34–210)	< 0.001
LDH (U/L)	572(337–861)	1000(896–2036)	< 0.001
Amylase (U/L)	142(94–204)	242(146–339)	< 0.001
Lipase (U/L)	149(79–272)	318(172–536)	< 0.001
BUN (mmol/L)	5.2(3.7–6.8)	7.3(5.2–13.0)	< 0.001
Creatinine (μmol/L)	71(61–91)	175(69–274)	< 0.001
Cystatin-C (mg/L)	1.2(0.9–1.5)	1.4(1.1–2.7)	< 0.001
Sodium (mmol/L)	135.3(132.0-138.0)	134.2(130.5-138.4)	0.468
Potassium (mmol/L)	3.5(3.3-4.0)	3.9(3.5–4.5)	< 0.001
CK (U/L)	259(97–804)	969(467–1942)	< 0.001
CK-MB (U/L)	25(14–41)	77(40–129)	< 0.001
Troponin I (pg/mL)	80.2(35.4-177.6)	233.5(101.6–407.0)	< 0.001
BNP (pg/mL)	60(22–158)	125(58–369)	0.001
PT (s)	11.3(10.6–12.0)	11.7(11.1–12.8)	0.003
INR	1.03(0.97–1.10)	1.07(1.01–1.18)	0.002
PTA (%)	99(88–111)	95(80–105)	0.038
APTT(s)	39.3(33.7–44.7)	52.7(44.1–63.1)	< 0.001
TT(s)	17.3(15.9–19.6)	22.4(19.2–28.5)	< 0.001
Fibrinogen (mg/dL)	256(206–300)	205(147–238)	< 0.001
D-dimer (ng/mL)	900(403–1992)	1862(826–3469)	< 0.001
CRP (mg/L)	6.9(3.0-13.2)	19.9(9.9–30.0)	< 0.001
Procalcitonin (ng/mL)	0.16(0.06–0.49)	0.91(0.27–1.96)	< 0.001
IL-6 (pg/mL)	21.9(14.2–36.5)	63.2(42.6-158.9)	< 0.001
SAA (mg/L)	33.5(22.5–71.4)	243.7(55.7-277.4)	< 0.001
ESR (mm/h)	8(5–14)	14(7–21)	0.003
Ferritin (ng/mL)	2947(2261–4435)	9371(4166–20,419)	< 0.001
Viral load (log_10_ copies/mL)	3.8(3.3–4.3)	5.4(4.4–6.1)	< 0.001

### Univariate and multivariate risk factors for in-hospital mortality of SFTS patients

By univariate analysis, male, age, diabetes, presence of abdominal pain, encephalopathy, SIRS, bacterial or fungal infections, AKI, ARDS and shock, serum ALT, AST, TBIL, ALP, GGT, LDH, amylase, lipase, BUN, creatinine, cystatin-C, potassium, CK, CK-MB, troponin I, BNP, PT, APTT, TT, CRP, procalcitonin, IL-6, SAA, ESR, ferritin levels, and viral load, low platelet count, hemoglobin, albumin, fibrinogen, and PTA were identified as risk factors for the prognosis of SFTS patients (Supplementary Table 2). These parameters were then subjected to collinearity analysis, male, age, diabetes, presence of SIRS, bacterial or fungal infections, encephalopathy, ARDS and shock, LDH, amylase, creatinine, troponin I, APTT, IL-6 and viral load were included in the multivariate analysis. The presence of encephalopathy (adjusted OR 5.584, 95% CI 2.322–13.428; *P* < 0.001), SIRS (adjusted OR 2.885, 95% CI 1.226–6.786; *P* = 0.005), bacterial or fungal infections (adjusted OR 2.249, 95% CI 1.157–5.710; *P* = 0.017), creatinine (adjusted OR 1.010, 95% CI 1.005–1.015; *P* < 0.001), IL-6 (adjusted OR 1.002, 95% CI 1.001–1.003; *P* = 0.040) were identified as independent predictors for the in-hospital mortality of SFTS patients on multivariate analysis (Table 2).

**Table 2:** Independent predictors of in-hospital mortality from multivariable logistic regression analyses

	Multivariable analysis	
	Adjusted OR (95% CI)	*P* value
Encephalopathy	5.584(2.322–13.428)	< 0.001
Bacterial or fungal infections	2.249(1.157–5.710)	0.017
SIRS	2.885(1.226–6.786)	0.005
Creatinine (μmol/L)	1.010(1.005–1.015)	< 0.001
IL-6 (pg/mL)	1.002(1.001–1.003)	0.040

### Demographics characteristics, comorbid conditions, clinical manifestations, laboratory test results, and cumulative survival rates of SFTS patients with and without SIRS

Compared with patients without SIRS, patients with SIRS had more presence of cough, sputum, anorexia, and encephalopathy. However, no significant differences were observed in the proportion of male, frequency of diabetes and hypertension, age, days from onset to admission, as well as the presence of headache, dizziness, chest distress, nausea, vomiting, abdominal pain, and diarrhea.

**Fig. 1:**
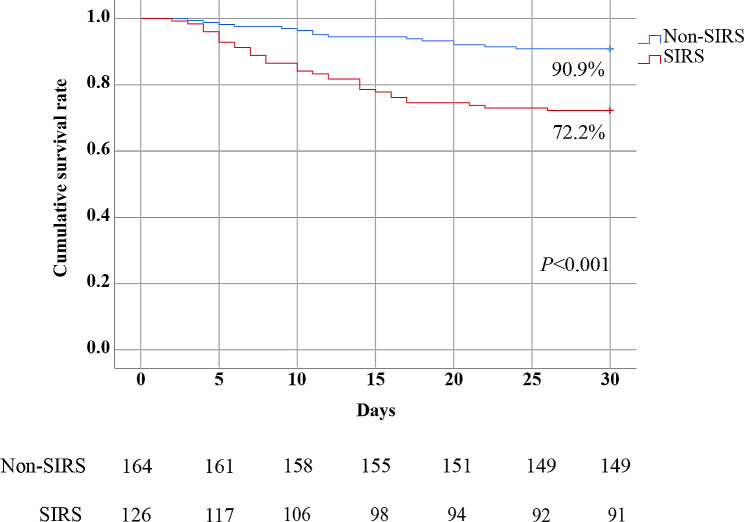
Kaplan–Meier curves show the cumulative survival rates of patients with SFTS in the non-SIRS and SIRS groups. A comparison of the survival estimates was performed using the Log-rank test, non-SIRS group vs. SIRS group, *P* < 0.001.

Among these laboratory parameters, WBC and neutrophils counts, fibrinogen levels of patients with SIRS were significantly lower than those of patients without SIRS. However, patients with SIRS had higher AST, LDH, amylase, lipase, CK, CK-MB, troponin I, APTT, TT, D-dimer, CRP, IL-6, SAA levels, and viral load than patients without SIRS. No significant differences were found between the two groups for the remaining variables (Table 3). The cumulative survival rate of patients with SIRS was significantly lower than that of patients without SIRS (72.2% vs. 90.9%, *P* < 0.001) (Fig. 1).

**Table 3:** Demographic characteristics, comorbid conditions, clinical symptoms, and laboratory parameters of SFTS patients with and without SIRS

	Non-SIRS (*n* = 164)	SIRS (*n* = 126)	*P* value
Male, n (%)	90(54.9)	57(45.2)	0.104
Age (years)	65(59–70)	66(58–69)	0.198
Diabetes, n (%)	14(8.5)	9(7.1)	0.663
Hypertension, n (%)	42(25.6)	29(23.0)	0.611
Days from onset to admission	6(5–7)	6(5–8)	0.864
Clinical manifestations, n (%)			
Neurological			
Headache	27(16.5)	28(22.2)	0.215
Dizziness	50(30.5)	45(35.7)	0.347
Encephalopathy	16(9.8)	29(23.0)	0.002
Respiratory			
Cough	30(18.3)	37(29.3)	0.027
Sputum	23(14.0)	36(28.6)	0.002
Chest distress	32(19.5)	27(21.4)	0.688
Gastrointestinal			
Anorexia	112(68.3)	100(79.4)	0.035
Nausea	110(67.1)	82(65.1)	0.722
Vomiting	52(31.7)	39(31.0)	0.891
Abdominal pain	25(15.2)	23(18.3)	0.494
Diarrhea	39(23.8)	31(24.6)	0.871
Laboratory parameters			
WBC (10^9^/L)	4.4(2.6–6.6)	2.9(1.7–5.4)	0.001
Neutrophils (10^9^ /L)	2.9(1.2–5.1)	1.8(1.0–4.0)	0.028
Neutrophils (%)	69.0(51.1–82.5)	69.1(57.1–81.8)	0.705
Lymphocyte (10^9^/L)	0.8(0.5–1.2)	0.6(0.4–1.1)	0.093
Lymphocyte (%)	19.8(11.4–32.8)	23.2(13.8–32.7)	0.331
Platelet (10^9^ /L)	43(29–63)	40(27–56)	0.061
Hemoglobin (g/L)	123 ± 18	124 ± 20	0.220
ALT (U/L)	73(47–117)	74(44–130)	0.868
AST (U/L)	149(67–369)	196(101–426)	0.017
TBIL(μmol/L)	11.4(8.1–17.4)	12.1(9.4–17.8)	0.221
Albumin (g/L)	30.0 ± 5.2	30.0 ± 4.4	0.788
Globulin (g/L)	26.1(23.4–29.6)	25.4(23.2–29.4)	0.298
ALP (U/L)	72(57–94)	76(57–111)	0.223
GGT (U/L)	41(24–88)	34(21–103)	0.920
LDH (U/L)	585(326–943)	715(449–1000)	0.013
Amylase (U/L)	146(95–220)	162(104–256)	0.044
Lipase (U/L)	149(79–280)	191(99–379)	0.013
BUN (mmol/L)	5.3(3.9-7.0)	5.4(3.8–8.1)	0.389
Creatinine (μmol/L)	71(61–96)	75(64–104)	0.063
Cystatin-C (mg/L)	1.2(0.9–1.5)	1.2(1.0-1.7)	0.125
Sodium (mmol/L)	135.7(132.6-138.2)	134.2(131.0-137.5)	0.117
Potassium (mmol/L)	3.6(3.3-4.0)	3.6(3.3–4.1)	0.560
CK (U/L)	251(89–853)	473(216–1127)	0.001
CK-MB (U/L)	26(13–43)	38(20–58)	0.001
Troponin I (pg/mL)	83.9(36.3-163.8)	132.1(42.8-317.5)	0.020
BNP (pg/mL)	65.6(19.6-169.2)	84.1(26.6-194.2)	0.327
PT (s)	11.3(10.6–12.0)	11.4(10.7–12.1)	0.660
INR	1.04(0.97–1.10)	1.04(0.99–1.12)	0.511
PTA (%)	98(88–109)	99(86–111)	0.877
APTT(s)	39.3(33.3–47.3)	42.2(36.8–51.2)	0.008
TT(s)	17.3(15.8–19.7)	18.4(16.2–22.1)	0.008
Fibrinogen (mg/dL)	260(200–312)	235(190–281)	0.010
D-dimer (ng/mL)	870(394–2195)	1247(610–2437)	0.034
CRP (mg/L)	4.2(2.8–9.8)	8.8(4.9–20.6)	0.015
Procalcitonin (ng/mL)	0.20(0.06–0.53)	0.26(0.09–0.91)	0.088
IL-6 (pg/mL)	22.1(14.2–39.3)	29.1(16.8–76.1)	0.001
SAA (mg/L)	34.5(23.6–74.9)	49.9(25.5-167.3)	0.004
ESR (mm/h)	8(5–14)	9(5–14)	0.623
Ferritin (ng/mL)	3028(2261–4957)	3589(2475–5786)	0.063
Viral load (log_10_ copies/mL)	3.8(3.4–4.4)	4.2(3.5–4.8)	0.002

### Prevalence and outcomes of different types of infections according to SIRS

The incidence of bacterial or fungal infections in patients with SIRS was significantly higher than those in patients without SIRS (74.6% vs. 49.4%, *P* < 0.001). According to the types of bacterial or fungal infections between patients with and without SIRS, both the incidence of pulmonary infections and multi-site infections were significantly higher in the SIRS group than those in the non-SIRS group (Table 4).

**Table 4:** Prevalence of different types of bacterial or fungal infections according to SIRS

	Non-SIRS (*n* = 164)	SIRS (*n* = 126)	*P* value
All infections, n (%)	81(49.4)	94(74.6)	< 0.001
Pulmonary infections, n (%)	55(33.5)	58(46.0)	0.031
Bacterial infections, n (%)	34(20.7)	47(37.3)	0.002
Fungal infections, n (%)	17(10.4)	9(7.1)	0.341
Bacterial and fungal coinfections, n (%)	4(2.4)	2(1.6)	0.613
Bloodstream infections, n (%)	3(1.8)	6(4.8)	0.153
Bacterial infections, n (%)	1(0.6)	2(1.6)	0.415
Fungal infections, n (%)	1(0.6)	1(0.8)	0.851
Bacterial and fungal coinfections, n (%)	1(0.6)	3(2.4)	0.200
Urinary tract infections, n (%)	7(4.3)	4(3.2)	0.629
Bacterial infections, n (%)	5(3.0)	2(1.6)	0.422
Fungal infections, n (%)	1(0.6)	1(0.8)	0.851
Bacterial and fungal coinfections, n (%)	1(0.6)	1(0.8)	0.851
Intestinal infections, n (%)	4(2.4)	6(4.8)	0.283
Bacterial infections, n (%)	1(0.6)	3(2.4)	0.200
Fungal infections, n (%)	2(1.2)	2(1.6)	0.790
Bacterial and fungal coinfections, n (%)	1(0.6)	1(0.8)	0.851
Intra-abdominal infections, n (%)	3(1.8)	4(3.2)	0.459
Bacterial infections, n (%)	1(0.6)	2(1.6)	0.415
Fungal infections, n (%)	1(0.6)	1(0.8)	0.851
Bacterial and fungal coinfections, n (%)	1(0.6)	1(0.8)	0.851
Multi-site infections, n (%)	9(5.5)	16(12.7)	0.030
Bacterial infections, n (%)	3(1.8)	4(3.2)	0.459
Fungal infections, n (%)	1(0.6)	2(1.6)	0.415
Bacterial and fungal coinfections, n (%)	5(3.0)	10(7.9)	0.053

**Fig. 2:**
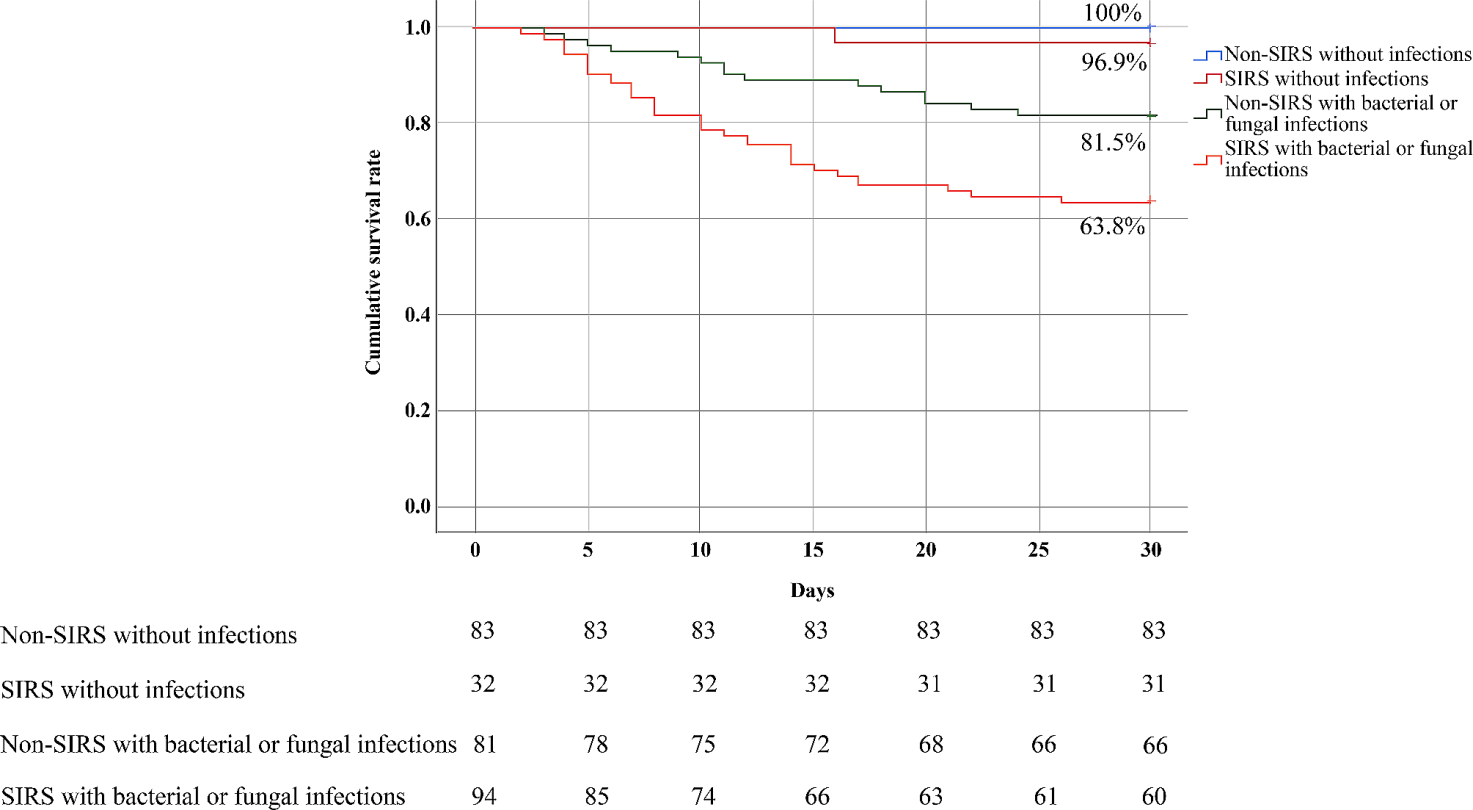
Kaplan–Meier curves show the cumulative survival rates of patients with SFTS based on the presence of SIRS and bacterial or fungal infections. A comparison of the survival estimates was performed using the Log-rank test, SIRS with bacterial or fungal infections group vs. non-SIRS with bacterial or fungal infections group, *P* = 0.008. SIRS with bacterial or fungal infections group vs. SIRS without bacterial or fungal infections group, *P* < 0.001. SIRS without infections group vs. non-SIRS without infections group, *P* = 0.107.

Patients were assigned into four groups according to the presence of bacterial or fungal infections and SIRS. Patients in the SIRS with bacterial or fungal infections group had a lower cumulative survival rate than patients in the SIRS without infections and non-SIRS with bacterial or fungal infections groups (63.8% vs.96.9%, *P* < 0.001; 63.8% vs. 81.5%, *P* = 0.008). However, there was no significant difference in the cumulative survival rates between the SIRS without infections and non-SIRS without infections groups (*P* = 0.107). It suggested that bacterial or fungal infections might predominantly contribute to high in-hospital mortality of SFTS patients with SIRS (Fig. 2).

## Discussion

In the present study, we found that almost half of patients with SFTS met SIRS criteria on admission and during hospitalization, which indicated that SIRS was a relatively frequent event in patients with SFTS. Furthermore, we revealed that SIRS was closely related to disease severity and high in-hospital mortality.

Several studies have reported that a variety of cytokines are generated during the acute onset of SFTSV infection, and they are associated with viral load and reflect the severity of the disease [17, 18]. Cytokines are a vital part of the immune system that act as couriers between cells, and participate in many pathological aspects of the inflammatory cascade resulting in SIRS [19]. Moreover, the intense interactions of cytokines leading to cytokine storms have been widely considered to be the main cause of a high mortality rate for some infectious diseases [20]. It implies that SIRS may be common and associated with poor prognosis in patients with SFTS. However, there is no study to report the incidence, clinical characteristics of SIRS, and its impact on the prognosis of patients with SFTS.

In the study, we showed that SIRS was more frequent in the non-survivor group than that in the survivor group, and revealed that SIRS was an independent risk factor for prognosis of patients with SFTS. We also demonstrated that patients with SIRS showed more presence of encephalopathy, and higher decreased levels of fibrinogen, as well as higher elevated levels of AST, LDH, amylase, lipase, CK, CK-MB, troponin I, APTT, TT, and D-dimer than patients without SIRS. These parameters are generally used to reflect the brain, liver, pancreas, heart, and coagulation functions, and their extremely abnormal levels represent severe organ damage, which usually appears in critically ill patients. Furthermore, we showed that the SFTSV viral load in patients with SIRS was significantly higher than that in patients without SIRS. Some studies have reported that vial load is an independent predictor of mortality in patients with SFTS [21, 22]. These results could be used to explain why patients with SIRS had a high in-hospital mortality. We also found that patients with SIRS had higher serum levels of CRP, IL-6, and SAA than patients without SIRS. They are acute-phase proteins and are involved in the process of initiating, sustaining, and regulating the inflammatory response [23, 24].

We observed an incidence of up to 43.4% of SIRS in patients hospitalized with SFTS. The prominent clinical features of patients with SFTS, namely fever and leukopenia, may increase the probability of diagnosing SIRS. Another reason for the frequent occurrence of SIRS in patients with SFTS could be explained by the high risk of bacterial or fungal infections development. A considerable number of studies have reported that patients with SFTS are more susceptible to pulmonary bacterial or fungal coinfections, which is associated with high mortality [25–27]. There is no study to explore the relationship between SIRS and bacterial or fungal infections in patients with SFTS. In the present study, we found that patients with SIRS had a significantly higher prevalence of pulmonary infections and multi-site infections than patients without SIRS. In addition, we revealed that the cumulative survival rate of patients in the SIRS with bacterial or fungal infections group was significantly lower than those of patients in the non-SIRS with bacterial or fungal infections and SIRS without infections groups. However, no significant differences were observed in the cumulative survival rate between the SIRS without infections and non-SIRS without infections groups. It suggested that bacterial or fungal infections might be an important factor contributing to the high in-hospital mortality of SFTS patients with SIRS. We also confirmed that both SIRS and bacterial or fungal infections were independent risk factors for the prognosis of patients with SFTS.

Though our study had a relatively larger sample size than some studies exploring the clinical characteristics and prognosis of patients with SFTS [28–30]. Certain limitations of our study need to be addressed. This was a retrospective single-center study, which might limit the interpretation of our findings. Additionally, it was difficult to interpret some components of SIRS in patients with SFTS. Actually, most of the patients with SFTS showed leukopenia and hyperthermia, which exposed us to the risk of also increasing false-positive diagnoses.

## Conclusions

In conclusion, this study systematically described detailed information on SIRS in SFTS, with high incidence and in-hospital mortality. It provides strong evidences to emphasize that patients with SFTS should be carefully monitored for the development of SIRS and corresponding measures should be taken to prevent it.

### Electronic supplementary material

Below is the link to the electronic supplementary material.

**Supplementary Material 1: Supplementary figure 1.** The study flow chart of the enrollment of patients. **Supplementary figure 2.** The epidemic curve of patients diagnosed with SFTS. **Supplementary table 1.** Number of patients with different diagnosis pattern of SIRS. **Supplementary table 2.** Predictors of in-hospital mortality from univariable logistic regression analyses

## Data Availability

All data generated or analysed during this study are included in this published article and its supplementary information files.

## List of abbreviations

AKI: Acute kidney injury
ALP: Alkaline phosphatase
ALT: Alanine aminotransferase
APTT: Activated partial thromboplastin time
ARDS: Acute respiratory distress syndrome
AST: Aspartate aminotransferase
BNP: Brain natriuretic peptide
BUN: Blood urea nitrogen
CK-MB: Creatinine kinase myocardial b fraction
CK: Creatinine kinase
CRP: C-reactive protein
ESR: Erythrocyte sedimentation rate
GGT: Gamma glutamyl transpeptidase
IL-6: Interleukin-6
INR: International normalized ratio
LDH: Lactate dehydrogenase
PT: Prothrombin time
PTA: Prothrombin activity
SAA: Serum amyloid A
SFTS: Severe fever with thrombocytopenia syndrome
TBIL: Total bilirubin
TT: Thrombin time
WBC: White blood cell

## References

[1] Kobayashi Y, Kato H, Yamagishi T (2020). Severe fever with Thrombocytopenia Syndrome, Japan, 2013–2017. Emerg Infect Dis.

[2] Lee HS, Kim J, Son K (2020). Phylogenetic analysis of severe fever with thrombocytopenia syndrome virus in Korean water deer (Hydropotes inermis argyropus) in the Republic of Korea. Ticks Tick Borne Dis.

[3] Yu XJ, Liang MF, Zhang SY (2011). Fever with thrombocytopenia associated with a novel bunyavirus in China. N Engl J Med.

[4] Moon J, Lee H, Jeon JH (2019). Aerosol transmission of severe fever with thrombocytopenia syndrome virus during resuscitation. Infect Control Hosp Epidemiol.

[5] Bao CJ, Guo XL, Qi X (2011). A family cluster of infections by a newly recognized Bunyavirus in Eastern China, 2007: further evidence of person-to-person transmission. Clin Infect Dis.

[6] Zhang X, Liu Y, Zhao L (2013). An emerging hemorrhagic fever in China caused by a novel bunyavirus SFTSV. Sci China Life Sci.

[7] Liu Q, He B, Huang SY (2014). Severe fever with thrombocytopenia syndrome, an emerging tick-borne zoonosis. Lancet Infect Dis.

[8] Li S, Li Y, Wang Q et al. Multiple organ involvement in severe fever with thrombocytopenia syndrome: an immunohistochemical finding in a fatal case. Virol J. 2018;15(1). 10.1186/s12985-018-1006-7 PMC5977472 29848330

[9] Wen H, Zhao L, Zhai S (2014). Severe fever with Thrombocytopenia Syndrome, Shandong Province, China, 2011. Emerg Infect Dis.

[10] Liu S, Chai C, Wang C (2014). Systematic review of severe fever with thrombocytopenia syndrome: virology, epidemiology, and clinical characteristics. Rev Med Virol.

[11] Balk RA (2013). Systemic inflammatory response syndrome (SIRS). Virulence.

[12] Sun Y, Jin C, Zhan F (2012). Host cytokine storm is Associated with Disease severity of severe fever with Thrombocytopenia Syndrome. J Infect Dis.

[13] Hu L, Wu T, Wang B et al. The regulation of seventeen inflammatory mediators are associated with patient outcomes in severe fever with thrombocytopenia syndrome. Sci Rep. 2018;8(1). 10.1038/s41598-017-18616-z PMC5760584 29317732

[14] Wang L, Zou Z, Ding K et al. Predictive risk score model for severe fever with thrombocytopenia syndrome mortality based on qSOFA and SIRS scoring system. BMC Infect Dis. 2020;20(1). 10.1186/s12879-020-05299-7 PMC7425036 32787952

[15] Bone RC, Balk RA, Cerra FB et al. Definitions for sepsis and organ failure and guidelines for the use of innovative therapies in sepsis. The ACCP/SCCM Consensus Conference Committee. American College of Chest Physicians/Society of Critical Care Medicine. Chest. 1992;101(6):1644–1655. 10.1378/chest.101.6.1644 1303622

[16] De Pauw B, Walsh TJ, Donnelly JP (2008). Revised definitions of invasive fungal disease from the European Organization for Research and Treatment of Cancer/Invasive Fungal Infections Cooperative Group and the National Institute of Allergy and Infectious Diseases Mycoses Study Group (EORTC/MSG) Consensus Group. Clin Infect Dis.

[17] Kwon J, Kim M, Kim JY (2018). Kinetics of viral load and cytokines in severe fever with thrombocytopenia syndrome. J Clin Virol.

[18] He Z, Wang B, Li Y (2021). Changes in peripheral blood cytokines in patients with severe fever with thrombocytopenia syndrome. J Med Virol.

[19] Matsuda N, Hattori Y (2006). Systemic inflammatory response syndrome (SIRS): Molecular Pathophysiology and Gene Therapy. J Pharmacol Sci.

[20] Karki R, Kanneganti T (2021). The ‘cytokine storm’: molecular mechanisms and therapeutic prospects. Trends Immunol.

[21] Wang Y, Song Z, Wei X (2022). Clinical laboratory parameters and fatality of severe fever with thrombocytopenia syndrome patients: a systematic review and meta-analysis. PLoS Negl Trop Dis.

[22] Liu M, Lei X, Yu X. Meta-analysis of the clinical and laboratory parameters of SFTS patients in China. Virol J. 2016;13(1). 10.1186/s12985-016-0661-9 PMC5129669 27899121

[23] Deutschman C (1998). Acute-phase responses and SIRS/MODS: the good, the bad, and the nebulous. Crit Care Med.

[24] De Jong HK, Tom VDP, Wiersinga WJ (2010). The systemic pro-inflammatory response in sepsis. J Innate Immun.

[25] Zhang Y, Huang Y, Xu Y (2022). Associated Microbiota and treatment of severe fever with thrombocytopenia syndrome complicated with infections. J Med Virol.

[26] Ge HH, Wang G, Guo PJ (2022). Coinfections in hospitalized patients with severe fever with thrombocytopenia syndrome: a retrospective study. J Med Virol.

[27] Zuo Y, Wang H, Huang J et al. Pulmonary infection in patients with severe fever with thrombocytopenia syndrome: a multicentre observational study. J Med Virol. 2023;95(4). 10.1002/jmv.28712 36991571

[28] Xiaowen X, Zhenlu S, Jingyu L et al. Analysis of clinical features and early warning indicators of death from severe fever with thrombocytopenia syndrome. Int J Infect Dis. 2018:S1149245146. 10.1016/j.ijid.2018.05.013 29859247

[29] Nie Q, Wang D, Ning Z (2020). Analysis of severe fever with Thrombocytopenia Syndrome in critical ill patients in Central China. Shock.

[30] He F, Zheng X, Zhang Z. Clinical features of severe fever with thrombocytopenia syndrome and analysis of risk factors for mortality. BMC Infect Dis. 2021,21(1). 10.1186/s12879-021-06946-3 PMC8669668 34906106

