# Supplementary material for: Systemic inflammatory response syndrome in patients with severe fever with thrombocytopenia syndrome: prevalence, characteristics, and impact on prognosis
Source: BMC Infect Dis. 2024 Jan 30;24:149. doi: 10.1186/s12879-024-09026-4 (PMC10829256; doi:10.1186/s12879-024-09026-4)
Supplement: Supplementary file 1 — Supplementary Material 1: Supplementary figure 1. The study flow chart of the enrollment of patients. Supplementary figure 2. The epidemic curve of patients diagnosed with SFTS. Supplementary table 1. Number of patients with different diagnosis pattern of SIRS. Supplementary table 2. Predictors of in-hospital mortality from univariable logistic regression analyses [file 12879_2024_9026_MOESM1_ESM.docx]

Supplementary figure 1. The study flow chart of the enrollment of patients.


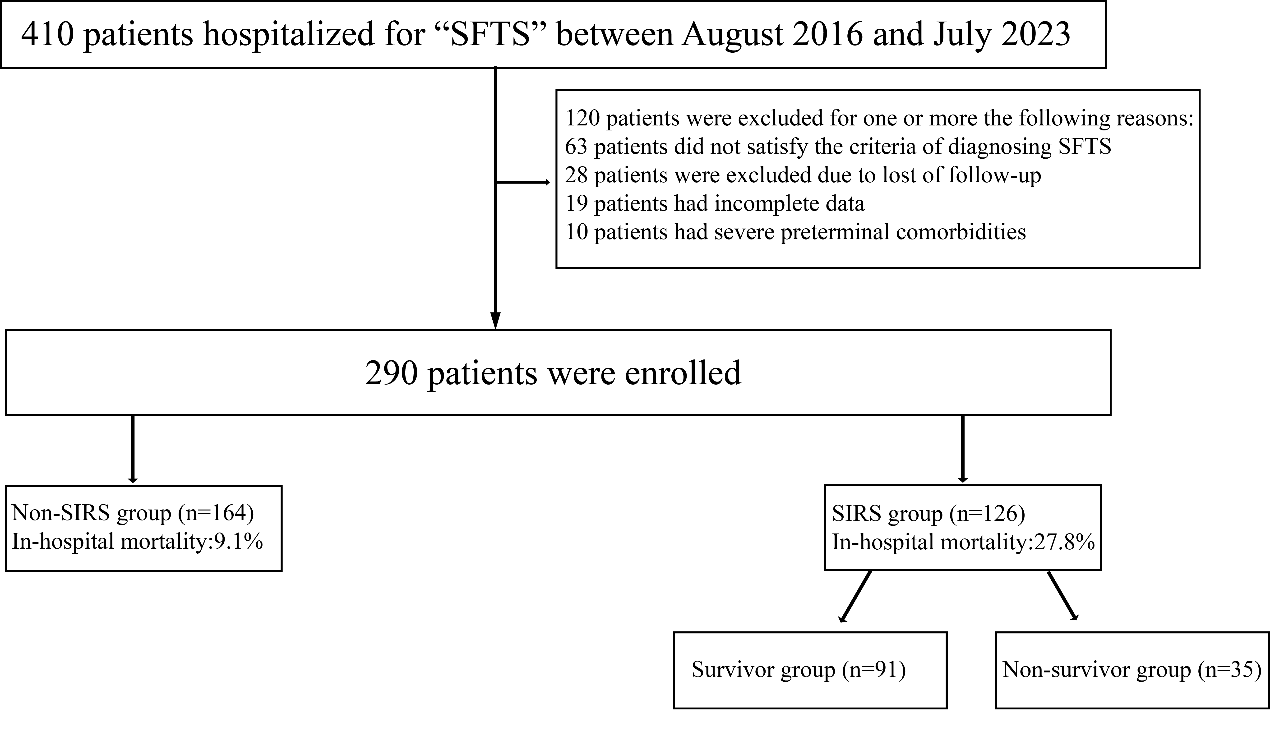


Supplementary figure 2. The epidemic curve of patients diagnosed with SFTS.


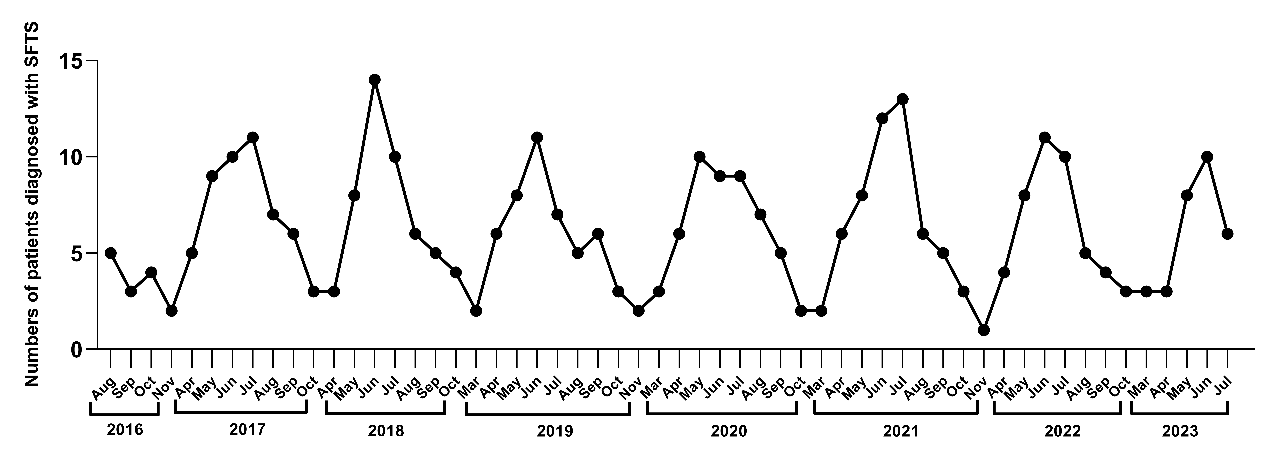


Supplementary table 1. Number of patients with different diagnosis pattern of SIRS.

| Diagnosis pattern of SIRS | Number of patients |
| --- | --- |
| 1+2 | 7 |
| 1+3 | 4 |
| 1+4 | 31 |
| 2+3 | 7 |
| 2+4 | 10 |
| 3+4 | 16 |
| 1+2+3 | 5 |
| 1+2+4 | 18 |
| 1+3+4 | 6 |
| 2+3+4 | 9 |
| 1+2+3+4 | 13 |

Note: (1) temperature of >38 ˚C or <36 ˚C; (2) heart rate of > 90 beats/minute; (3) respiratory rate of > 20 breaths/minute; (4) white blood cell (WBC) count > 12,000/mm^3^ or < 4000/mm^3^, or differential count > 10% immature polymorphonuclear neutrophil cells.

Supplementary table 2. Predictors of in-hospital mortality from univariable logistic regression analyses.

|  | Univariable analysis | |
| --- | --- | --- |
|  | Crude OR (95% CI) | *P* value |
| Male | 1.865(0.998-3.483) | 0.051 |
| Age(years) | 1.039(1.001-1.078) | 0.044 |
| Diabetes | 2.279(0.885-5.871) | 0.088 |
| Hypertension | 1.249(0.629-2.477) | 0.525 |
| Bacterial or fungal infections | 3.821(1.977-7.382) | <0.001 |
| Days from onset to admission | 1.013(0.916-1.120) | 0.802 |
| Clinical manifestations |  | |
| Neurological |  | |
| Headache | 1.258(0.896-1.542) | 0.674 |
| Dizziness | 1.102(0.914-1.256) | 0.586 |
| Encephalopathy | 9.626(4.719-19.638) | <0.001 |
| Respiratory |  | |
| Cough | 2.115(0.894-2.548) | 0.783 |
| Sputum | 1.672(0.928-4.214) | 0.256 |
| Chest distress | 1.458(0.882-2.746) | 0.425 |
| Gastrointestinal |  | |
| Anorexia | 1.325(0.906-2.362) | 0.812 |
| Nausea | 0.985(0.886-1.074) | 0.673 |
| Vomiting | 1.205(0.913-1.650) | 0.524 |
| Abdominal pain | 7.326(3.662-14.656) | <0.001 |
| Diarrhea | 0.936(0.894-1.382) | 0.254 |
| SIRS | 4.255(2.199-8.324) | <0.001 |
| ARDS | 6.164(4.162-9.527) | <0.001 |
| AKI | 9.257(4.158-16.465) | <0.001 |
| Shock | 10.152(6.524-17.846) | <0.001 |
| Laboratory parameters |  | |
| WBC (10^9^/L) | 0.979(0.897-1.068) | 0.628 |
| Neutrophils (10^9^ /L) | 0.970(0.876-1.073) | 0.552 |
| Neutrophils (%) | 1.000(0.994-1.006) | 0.953 |
| Lymphocyte (10^9^/L) | 1.061(0.697-1.613) | 0.783 |
| Lymphocyte (%) | 0.988(0.966-1.010) | 0.289 |
| Platelet (10^9^ /L) | 0.976(0.961-0.992) | 0.003 |
| Hemoglobin (g/L) | 0.989(0.974-1.004) | 0.153 |
| ALT (U/L) | 1.003(1.001-1.006) | 0.007 |
| AST (U/L) | 1.002(1.001-1.003) | <0.001 |
| TBIL (μmol/L) | 1.011(0.997-1.024) | 0.115 |
| Albumin (g/L) | 0.851(0.787-0.921) | <0.001 |
| Globulin (g/L) | 1.014(0.954-1.079) | 0.651 |
| ALP (U/L) | 1.007(1.003-1.010) | <0.001 |
| GGT (U/L) | 1.003(1.001-1.005) | 0.002 |
| LDH (U/L) | 1.002(1.001-1.002) | <0.001 |
| Amylase (U/L) | 1.003(1.001-1.005) | 0.001 |
| Lipase (U/L) | 1.002(1.001-1.003) | 0.001 |
| BUN (mmol/L) | 1.153(1.087-1.224) | <0.001 |
| Creatinine (μmol/L) | 1.014(1.010-1.019) | <0.001 |
| Cystatin-C (mg/L) | 3.080(1.995-4.753) | <0.001 |
| Sodium (mmol/L) | 0.996(0.942-1.053) | 0.897 |
| Potassium (mmol/L) | 3.090(1.842-5.185) | <0.001 |
| CK (U/L) | 1.001(1.001-1.002) | 0.004 |
| CK-MB (U/L) | 1.012(1.007-1.018) | <0.001 |
| Troponin I (pg/mL) | 1.001(1.001-1.002) | 0.027 |
| BNP (pg/mL) | 1.001(0.998-1.002) | 0.135 |
| PT (s) | 1.009(0.964-1.057) | 0.695 |
| INR | 1.003(0.990-1.017) | 0.631 |
| PTA (%) | 0.980(0.963-0.998) | 0.026 |
| APTT (s) | 1.080(1.050-1.110) | <0.001 |
| TT (s) | 1.107(1.050-1.167) | <0.001 |
| Fibrinogen (mg/dL) | 0.987(0.982-0.993) | <0.001 |
| D-dimer (ng/mL) | 1.002(0.985-1.004) | 0.105 |
| CRP (mg/L) | 1.027(1.013-1.042) | <0.001 |
| Procalcitonin (ng/mL) | 1.041(1.001-1.083) | 0.043 |
| IL-6 (pg/mL) | 1.002(1.001-1.003) | <0.001 |
| SAA (mg/L) | 1.013(1.010-1.017) | 0.001 |
| ESR (mm/h) | 1.023(1.002-1.045) | 0.030 |
| Ferritin (ng/mL) | 1.001(1.001-1.002) | <0.001 |
| Viral load (log_10_ copies/mL) | 5.151(3.318-7.995) | <0.001 |
